# Study Design, Statistical Method, and Level of Evidence in Japanese and American Clinical Journals

**DOI:** 10.2188/jea.12.266

**Published:** 2007-11-30

**Authors:** Tsuguya Fukui, Mahbubur Rahman, Miho Sekimoto, Kenji Hira, Kenji Maeda, Takeshi Morimoto, Masashi Goto, Hidehiko Suzuki, Takuro Shimbo

**Affiliations:** Department of General Medicine and Clinical Epidemiology, Kyoto University Graduate School of Medicine, Kyoto University Hospital.

**Keywords:** USA, Japan, statistical procedures, medical journals, study design

## Abstract

Clinical articles published in Japanese journals are said to be characterized by poor study design, less sophisticated statistics, and producing few high-grade clinical evidences. Two American and two Japanese medical journals, published in 1990, 1993, 1996, and 1999 were compared to find out the differences regarding study design, statistical methods, and level of clinical evidence of original articles and synthetic studies. There were 1689 original articles in American and 308 in Japanese journals. Regarding study design, American articles contributed much more to randomized controlled trials/controlled trials/clinical trials (27.9% vs. 14.3%, p=0.001), cohort studies (21.6% vs. 6.2%, p=0.001), and case-control studies (6.5% vs.0.3 %, p=0.000). Among original articles in American and Japanese journals, mean number of statistical methods used were 2.4 and 1.7 per article (p=0.000), respectively. Articles providing high grade clinical evidence (grade Ia, Ib & IIa) were much greater in proportion in American journals than Japanese journals (31.1% vs. 12.7%, p=0.001). The overall picture of Japanese medical articles seems to be improving recently, at least in terms of statistical methods toward more diversified and sophisticated way of use, compared to the previous data.

## BACKGROUND

Original or synthetic articles containing high grade clinical evidence have been extending a great impact to the clinicians worldwide. Quality of clinical study is mostly determined by study design. Among a variety of study designs, randomized controlled trial (RCT) is generally considered far most superior to others in providing the least biased results. In addition to study design, no one now doubts the importance of statistics as an armamentarium in modem scientific research. In fact a variety of statistical procedures have been widely used and made great contributions to scientific progresses by enhancing the quality of research through compactly summarizing data and withdrawing valuable inferences. Medicine is one of these scientific branches which owe their tremendous achievements to statistics in the past several decades.

However, it has been said that statistical methods used by the Japanese researchers in clinical medicine were disproportionately limited in number and kind compared with those by the western counterparts. For example, it is reported that the majority of statistical methods used in the journals published in Japan were either t -tests or several other simple procedures^1^^)^. In contrast, the majority of those used by American journals were much more complex and sophisticated in kind.

The medical literature is vast and rapidly expanding and the readers are overwhelmed by the articles. To find out high quality articles worth reading and quoting, a scale for rating the grade of evidence to rank the strength of the findings and recommendations is helpful, such as the one developed by the U. S. Preventive Services Task Force^2^^)^. Journals are thus showing more interest in publishing articles containing high grade of evidence.

We conducted this comparative study to describe the current status of study design employed, statistical methods used, and also to quantify the grade of clinical evidence in articles published in American and Japanese clinical journals.

## MATERIALS AND METHODS

We selected two medical journals each from the USA and Japan to obtain the data on study design and statistical methods used according to the following criteria; the journals should (1) relate to clinical medicine, (2) use English, (3) circulate in high volume in each country, and (4) come out at least on bimonthly basis. Based on these criteria, we selected “New England Journal of Medicine (NEJM)” and “Journal of American Medical Association (JAMA)” from the USA among the 105 journals listed in “Medicine, General and Internal category” set by Institute for Scientific Information (ISI)^3^^)^. “Internal Medicine (IM)” and “Japanese Heart Journal (JHJ)” were selected from Japan. There were many other options regarding American journals. But we chose NEJM and JAMA, because they had highest impact factors in the year 2000 among the journals in “Medicine, General and Internal category” and they enjoy a high circulation worldwide including Japan. On the other hand, options for Japanese journals were very few. We picked up IM, the sole Japanese journal from “Medicine, General and Internal category” and JHJ, another highly circulated clinical journal in English.

Articles chosen for analysis here were original papers published in these journals during 1990, 1993, 1996, and 1999. They involved at least ten patients or presented experimental studies with at least 10 measurements or numeric data of any kind. Besides, such synthetic studies as meta-analysis, cost-effectiveness analysis and decision analysis, were also included. Years of publication (1990, 1993,1996, and 1999) with uniform interval were selected to see the trend of study design, statistical method and grade of evidence. Data were withdrawn from each original article in terms of study design (e.g., randomized controlled trials, other clinical trials, case-control study, cohort study, case series, cross sectional study and synthetic studies), type and number of statistical procedure used, and grade of evidence (Table 1) according to the reported classification^2^^)^, and the number and type of statistical methods used. Number of statistical methods used was based on a simple count of statistical tests used, not on broad categories of statistical procedures. Classification of study design was based on most common classification used in clinical research. Seven medical doctors experienced in clinical epidemiology and biostatistics abstracted the data for this study.

**Table 1.** Grade of evidence formulated by the U. S. Preventive Services Task Force^2^^)^.

Grade Ia	: Evidence obtained from meta-analysis of RCT
Grade Ib	: Evidence obtained from RCT
Grade IIa	: Evidence obtained from controlled study without randomization
Grade IIb	: Evidence obtained from quasi-experimental study
Grade III	: Evidence obtained from well designed non-experimental descriptive studies, such as comparative studies, correlation studies and case-control studies
Grade IV	: Evidence obtained from expert committee reports or opinions and /or clinical experience of respected authorities

### Statistical Analyses

Statistical analysis included descriptive statistics and bivariate analysis. We used STATA software^4^^)^. Categorical data were contrasted using the chi-square test; when the expected value of a cell was less than 5, the Fisher exact test was used. To compare continuous data, the t test was used for normally distributed variables; otherwise, the Mann Whitney U test was used. Mantel extension test and nonparametric test for trend were used to see the trends of different parameters over time. All tests of significance were two-tailed and values of P<0.05 were considered significant.

## RESULTS

Among original clinical articles subjected to current analysis, a total of 1689 were from two American journals and 308 from two Japanese journals in the years 1990, 1993, 1996, and 1999. American journals contributed more in respect to randomized controlled trials/controlled trials/clinical trials (27.9% vs. 14.3%, p=0.001), cohort studies (21.6% vs. 6.2%, p=0.001), case-control studies (6.5% vs. 0.3%, p=0.001) and synthetic studies (3.0% vs. 0%, P=0.001) (Table 2). However, regarding case series, Japanese journals had higher proportion of articles than that of American journals (39.6% vs. 12.3%, p=0.001) (Table 2). There was no difference in study design between the two Japanese journals (p=0.97). However, significant difference was observed between the two American journals (p=0.001).

**Table 2.** Comparison of study designs between American and Japanese journals.

	Number of articles in American journals (%)	Number of articles in Japanese journals (%)
Total Number of original articles including synthetic studies	1689	308
RCT/Controlled Trial/Clinical Trial	471 (27.9)	44 (14.3)
Cohort studies	364 (21.6)	19 ( 6.2)
Case-control studies	110 ( 6.5)	1 (0.03)
Case series	208 (12.3)	122 (39.6)
Synthetic studies (decision analysis, cost-effectiveness analysis, meta-analysis)	51 ( 3.0)	0 (0)
Cross sectional/Observational/Survey studies	426 (25.2)	98 (31.8)
Others	59 ( 3.5)	24 ( 7.8)

RCT = Randomized Controlled Trial

American journals = NEJM, JAMA

Japanese journals = IM, JHJ

With respect to the articles with no statistical method used there was no significant difference between the American and Japanese journals (17.5% vs. 13.2%, p=0.43). American journals used multiple statistical methods more frequently than those of Japanese journals (67.0% vs. 54.2%, p=0.001). The mean number of statistical methods used per article was 2.4 for the American and 1.7 for Japanese journals (p=0.001). Even after adjusting by study design, significant difference persisted (2.3 in American journals Vs. 1.8 in Japanese journals). There was no difference in the mean number of statistics between the two Japanese journals (IM 1.6 JHJ 1.7, p=0.43). However, significant difference was observed between the two American journals (NEJM 2.6 JAMA 2.1, p=0.001). Articles from Japan which were published in the above-mentioned American journals had 2.8 statistical methods per article, greater than that of average values of American and Japanese journals.

Articles having higher grade of evidence (Grade Ia,Ib and IIa) were also found to be more in American journals (31.1% vs. 12.7%, p=0.001) (Table 3). There was no difference in generating higher grade of evidence between the two Japanese journals (p=0.51). However, significant difference was observed between the two American journals, JAMA producing more grade Ia evidence (p=0.001), while NEJM grade Ib& IIa (p=0.01).

**Table 3.** Grade of clinical evidence found in American and Japanese journals.

Grade of evidence	Articles containing evidence	

	American journals	Japanese journals
	(N=1,689)	(N=308)
Grade Ia	17 ( 1.0%)	0 ( 0 %)
Grade Ib	388 (23.0%)	5 ( 1.6%)
Grade IIa	120 ( 7.1%)	34 (11.0%)
Grade IIb	145 ( 8.6%)	34 (11.0%)

Articles having Grade III &IV evidence are not tabulated.

**Figure 1.**
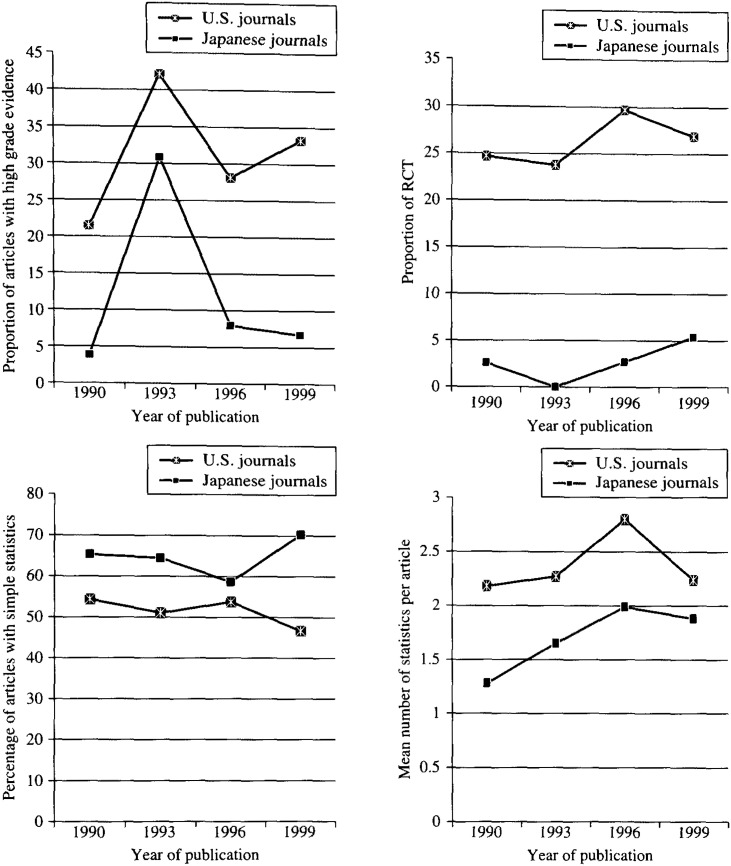
Comparison of American and Japanese journals regarding proportion of articles with high grade evidences (grade Ia, Ib, and IIa), proportion of RCT, percentage of articles with simple statistics (t test, chi-square test, and correlation coefficient), and mean number of statistics.

Figure 1 shows the trends of different parameters for American and Japanese journals over the period of 1990 through 1999. Only positive trend in the mean number of statistics was found to be significant (p=0.01) for Japanese, while for American journals, in addition to the mean number of statistics (p=0.048), positive trend was also found in the proportion of higher grade of evidences (grade Ia, Ib, and IIa) (p=0.01).

## DISCUSSION

Our results showed quite clearly that the original articles published in the Japanese journals much less contributed to randomized controlled or case-control studies and that they employed smaller number of and less sophisticated statistical methods compared with those in the American journals. There are several possible reasons behind these findings.

Firstly, the difference in the use of statistical methods may well be the mere reflection of the difference in study design. However, even after adjusting study design, the difference in the mean number of statistics remained almost similar. The number and kind of statistical methods employed in research articles are determined not only by study design but also by training background of clinicians and inclusion of biostatician/clinical epidemiologist in the research team. The majority of research topics for Japanese researchers might have essential characteristics to be conducted by one of the simpler and less costly study designs other than randomized or case-control studies. Then, it’s natural for these Japanese journals, in which the majority of authors were Japanese researchers, to employ smaller number of and less sophisticated statistical methods to analyze the data obtained by these simpler and less costly study designs. In this line, the resource and manpower may not be adequately prepared for the researches which require sophisticated study design and statistical approach. It is rare for academic institutions in Japan to have clinical research teams involving a biostatistician and /or a clinical epidemiologist as formal staffs. Lack of cooperation of clinicians with biostatistician and/or a clinical epidemiologist could lead to the study design of lower grade and the use of simpler statistics.

Secondly, the level of training in biostatistics may not be adequate on the part of Japanese physicians. For example, Tango pointed out in his recent article that the description of the study design, sampling procedures and specific statistical methods used were not adequate in the articles by Japanese researchers^1^^)^. This situation can only be improved by better educating clinical researchers and ideally all medical students in principles of biostatistics and clinical epidemiology. The curriculum of clinical epidemiology proposed by Japan Society for Medical Education will be of help in this regard^5^^)^.

A hopeful sign, however, emerges when the current results are compared to those reported by Tango in 1981. He showed that 97% of the statistical methods used in Japanese medical journals were bounded by three statistical tests: t-test, chi-square test, and correlation coefficient analysis^6^^)^. The proportion of articles with the statistical methods limited to these three tests was 65.4%, 65.3%, 58.7%, and 70.3% in 1990, 1993, 1996, and 1999, respectively. Positive trend was also found in terms of the mean number of statistical procedures per article (p=0.01). Furthermore, the current list of the statistical procedures as shown in this study is much longer and diversified. Even in American journals in early 1970s t-test was the most common statistical procedure^7^^)^. Greater number of randomized controlled trials and case-control studies is clearly responsible for the diversity of the statistical procedures used by the American researchers.

In the context of evidence-based medicine widely accepted internationally nowadays, clinical studies in Japan will inevitably be geared toward randomized controlled studies which ultimately provide less biased, statistically robust results and first grade evidences.
